# Multi-Omics Profiling Identifies Apolipoprotein E as an Important Regulator of Steroidogenesis in Bactrian Camel Poll Glands During the Breeding Season

**DOI:** 10.3390/ani15213147

**Published:** 2025-10-30

**Authors:** Qi Ma, Bohao Zhang, Jianfu Li, Quanwei Zhang

**Affiliations:** 1 College of Life Science and Technology, Gansu Agriculture University, Lanzhou 730070, China; maq@gsau.edu.cn (Q.M.); 2 College of Veterinary Medicine, Gansu Agriculture University, Lanzhou 730070, China; zhangbhgs@163.com (B.Z.); 1073325140048@st.gsau.edu.cn (J.L.); 3 Gansu Key Laboratory of Animal Generational Physiology and Reproductive Regulation, Lanzhou 730070, China

**Keywords:** male Bactrian camel, poll gland, steroid hormone, APOE, cholesterol, multi-omics, reproduction

## Abstract

**Simple Summary:**

The poll gland of male Bactrian camels plays a significant role in their reproductive system by secreting substances that influence female estrus and mating behaviors. However, the mechanisms behind these processes remain poorly understood. This study reveals how cholesterol and steroid hormones are regulated in the poll gland during the breeding season. An analysis of metabolites and proteins in the gland identified several molecules that are crucial for steroid hormone synthesis. Among them, apolipoprotein E (APOE) was identified as a key regulator of cholesterol metabolism, which is essential for the production of steroid hormones such as testosterone. The study revealed that APOE and its related proteins interact with androgen receptors to affect hormone synthesis in the poll gland. By offering valuable insights into the biochemical processes involved in camel reproduction, this study can contribute to improving camel breeding practices.

**Abstract:**

Camel poll gland tissues (PGs) secrete amber liquid and volatile substances during the breeding season, inducing estrus and mating in female camels. These processes are mainly regulated by steroid hormones and their receptors, including the Androgen Receptor (AR). However, the functional components of PGs and their regulatory mechanisms in camel reproduction remain unclear. Therefore, in this study, we identified candidate differentially expressed metabolites (DEMs) and differentially expressed proteins (DEPs) associated with steroids through a multi-omics analysis of PGs during the male camel breeding season. We found that total cholesterol and testosterone concentrations were significantly increased in camel PGs at different stages of the breeding season. DEMs and DEPs related to cholesterol or steroids were analyzed using metabolomics and data-independent acquisition proteomics in the PGs of male Bactrian camels at different stages (early and peak breeding seasons), and the potential mechanism of steroid hormone synthesis was further explored. The metabolomics results identified 13 DEMs related to steroids in PGs at different stages. The proteomics results revealed seven GO terms and 69 DEPs related to steroids, with apolipoprotein E (APOE) identified as the core DEP. Pathway analysis confirmed that APOE and related DEPs were involved in cholesterol and steroid hormone synthesis. Immunostaining showed that APOE and AR were co-localized in the cytoplasm of acinar epithelial cells, and exhibited opposite expression trends in PGs during different breeding stages. These findings demonstrate that APOE- and AR-mediated cholesterol metabolism plays an important role in steroid hormone synthesis during camel reproductive activity, providing valuable insights into the mechanisms of steroid synthesis in PGs. This study offers a theoretical framework for understanding camel reproductive biology, particularly the interplay between APOE and AR in regulating cholesterol metabolism and steroidogenesis.

## 1. Introduction

The Bactrian camel (*Camelus bactrianus*), a member of the genus Camelus in the family Camelidae, is mainly distributed in the arid and semi-arid ecosystems of Africa and western Asia [1]. Camels also represent the main livestock in the arid and semi-arid desert areas of northwest China. Because of their unique body structure, metabolic characteristics, and long-term evolutionary adaptations, camels have developed stress resistance, including tolerance to heat, cold, hunger, thirst, sandstorms, and epidemic diseases [2]. Camels also exhibit unique reproductive physiological mechanisms. The breeding season of male camels begins in mid-November and lasts approximately four months, with camels becoming anestrus in mid-March of the following year. Despite their long breeding cycle, reproductive performance is lower than that of other livestock, such as cows and pigs, because of slow growth and development, long generation intervals, and prolonged gestation [3,4].

Male camels possess special secretory glands, known as poll gland tissues (PGs), located under the skin on both sides of the first poll vertebra behind the occipital ridge-poll glands [+]. These glands, also known as “occipital glands,” exist only in fertile male camels and exhibit an inverted “V” shape, comprising several almond-colored, pyramid-shaped lobules [5]. PGs enhance amber secretion during breeding season. The color of these secretions changes to dark brown or black upon exposure to air. The main components of PG secretions include steroid hormones and short-chain fatty acids, which are similar to sexual pheromones and may stimulate or induce estrus and mating in female camels through odor [6,7].

Steroid hormones are tetracyclic aliphatic hydrocarbon compounds with a cyclopentane polyhydrophenanthrene core. These hormones, predominantly synthesized in the smooth endoplasmic reticulum and mitochondria of testicular interstitial and adrenocortical cells, play important roles in animal reproduction, physiology, and pathology [8]. The synthesis of steroid hormones (including testosterone, estradiol, and corticosteroids) is precisely regulated by the hypothalamus–pituitary–gonadal axis through both acute and chronic regulation [9,10]. Acute synthesis mainly involves the transport of cholesterol from the outer mitochondrial membrane to the inner membrane of the cell and is regulated by acute regulatory proteins involved in steroid hormone synthesis [11]. Under normal physiological conditions, chronic regulation involves steroid hormone synthesis in the gonads, adrenal glands, and other tissues via cholesterol transport. Cholesterol is the only precursor for steroid hormone synthesis, regulating the content and biological activity of steroid hormones in the body through a self-metabolic balance [12]. In mitochondria, cholesterol is converted to pregnenolone by cholesterol side-chain cleavage enzyme, followed by transportation to the endoplasmic reticulum, where it is converted to testosterone and dihydrotestosterone by 3β-hydroxysteroid dehydrogenase (HSD3β) [13]. HSD17β3 is considered the key enzyme for testosterone production, although HSD17β11 can also catalyze the conversion of 5α-androstane to androgens. The metabolic balance of cholesterol is closely related to steroid hormone levels in mammals. Thus, clarifying the relationship between cholesterol metabolism and steroid hormone synthesis will provide insights into the mechanisms of animal reproductive regulation.

Previous studies have found that male camels secrete sex hormone-like substances from PGs during the breeding season, thereby inducing female estrus and mating [14,15,16]. Cellular cholesterol is important for the synthesis of steroid hormones. However, the specific molecular mechanisms by which the poll gland regulates cholesterol metabolism and steroid hormone biosynthesis during the breeding season remain poorly understood, especially in relation to the reproductive inefficiency observed in camels.

We hypothesize that key metabolic and protein factors in the poll gland, particularly those involved in cholesterol transport and metabolism, play crucial roles in regulating steroid hormone synthesis during the breeding season. Accordingly, the aim of this study was to identify candidate differentially expressed metabolites (DEMs) and differentially expressed proteins (DEPs) associated with steroids through a multi-omics analysis of PGs during the male camel breeding season. Combined with the steroid hormone synthesis process, the role and mechanisms of candidate targets in steroid hormone synthesis were speculated and verified through molecular biological experiments. These findings help elucidate the molecular mechanisms of steroid hormone synthesis in camel PGs and provide a theoretical basis for improving the reproductive capacity of camels.

## 2. Materials and Methods

### 2.1. Sample Collection and Processing

Male Bactrian camels (eight years old, 500 ± 10 kg), with similar physiological conditions and no obvious reproductive diseases, were free herded in semi-desert areas of Zhangye city (Gansu Province, China) during the rutting season (mid-November to mid-January of the following year). Camels with no obvious PG secretions but exhibiting rutting behaviors, such as tail-urine flapping and defecation display, rubbing the anterior surface of the hump with the poll gland, and competing for females, as described previously [17], were selected as the early breeding season (control) group in mid-November. Camels showing frequent tail-urine flapping and defecation behavior and abundant PG secretions were selected as the peak breeding season (experimental) in mid-January of the following year. Fresh PGs from the two groups were immediately collected and either stored in liquid nitrogen or fixed with 4% paraformaldehyde after slaughter in a commercial slaughterhouse, as previously described [18]. Owing to ethical and logistical constraints in sampling male Bactrian camels, three independent animals were included in each group, for histological sectioning and gene/protein expression analysis (frozen and fixed samples, *n* = 3). For proteomic analysis, three independent animals per group were also used (*n* = 3), while for metabolomic analysis, six independent animals per group were included (*n* = 6), meeting the minimum requirement for biological reproducibility. This study was approved by the Animal Protection Committee of Gansu Agricultural University Laboratory Animal Ethics (code: GSU-LC-2020-39).

### 2.2. Total Cholesterol and Testosterone Measurement

Total cholesterol (T-CHO) and testosterone in the PGs of control and experimental groups were measured using a cholesterol detection kit (Jiancheng, Nanjing, China) and iodine testosterone radioimmunoassay kit (Furui Runze, Beijing, China), respectively, according to the manufacturer’s instructions [19,20]. All assays were performed at least in triplicate.

### 2.3. Metabolite Sequencing

PGs (100 mg) were homogenized in 200 μL H_2_O with five ceramic beads using a homogenizer (Wonbio, Shanghai, China). A methanol/acetonitrile (1:1, *v*/*v*) solution (800 μL) was added to the homogenate for metabolite extraction. After centrifugation, the supernatant was dried in a vacuum centrifuge (Bio-Rad, Hercules, CA, USA). The dried extract was dissolved in an acetonitrile/water (1:1, *v*/*v*) solvent for liquid chromatography–mass spectrometry analysis in positive and negative ion modes. The analysis was performed using UHPLC (1290 Infinity LC, Agilent Technologies, Santa Clara, CA, USA) coupled with mass spectrometry (AB Sciex TripleTOF 6600, Waters, Milford, MA, USA). Quality control samples were prepared by combining equal volumes of the extraction solutions from all samples. Chromatographic separation was performed using an ACQUITY UPLC HSS T3 column (Waters, Milford, MA, USA). Liquid chromatography–mass spectrometry analysis was conducted on an ACQUITY UPLC HSS T3 column (100 × 2.1 mm, 1.8 μm; Waters) with a flow rate of 0.3 mL/min. The mobile phases consisted of water (A) and acetonitrile (B), each containing 0.1% formic acid. The gradient elution lasted 18 min. Mass spectrometry was performed using an AB Sciex TripleTOF 6600 with electrospray ionization in both positive and negative modes. Liquid chromatography–mass spectrometry data were processed using Progenesis QI software (Nonlinear Dynamics, Newcastle, UK) to extract raw peaks, filter and calibrate the baseline, align peaks, deconvolute signals, identify peaks, and integrate peak areas. Qualitative analysis of metabolites was performed using the Human Metabolome Database. Candidate DEMs were screened under the following thresholds: false discovery rate < 0.05 and variable importance in projection > 1, where the latter value represents the contribution of each metabolite to the model and is commonly used for variable selection in partial least squares discriminant analysis [21]. The identified DEMs were annotated using the Kyoto Encyclopedia of Genes and Genomes (KEGG) compound database (https://www.kegg.jp/, accessed on 10 June 2025). Annotated metabolites were then mapped to the KEGG pathway database. In addition, functional annotation was compared with the Gene Ontology (GO) database (https://geneontology.org/, accessed on 10 June 2025) and available camel genomic resources (Camelus_bactrianus_GCF_000767855.1 in NCBI) to ensure annotation accuracy.

### 2.4. Bioinformatic Analysis of Data-Independent Acquisition (DIA) Proteomics

Protein extraction, liquid chromatography separation, mass spectrometry, and proteomic data analysis of PGs from the control and experimental groups were performed as previously described [22]. The raw DIA proteomic data are deposited in ProteomeXchange under accession number PXD047457. Compared to the control group, significant DEPs in the experimental group were identified with an absolute fold change >1.50 and *p*.adjust < 0.05 using R packages (v4.3.1; https://www.r-project.org/, accessed on 25 July 2025). Data processing and visualization were performed with commonly used R packages, including *limma* (v3.58.1), *ggplot2* (v3.4.4), and *clusterProfiler* (v4.10.0). GO and KEGG pathway enrichment analyses were performed to determine the functions of the DEPs, as described previously [23]. GO terms and KEGG pathways with *p*.adjust < 0.05 were considered statistically significant. GO terms with *p* < 0.05, and *p*.adjust < 0.05, including DEPs associated with steroids, were then selected for functional analysis. Pathways involving apolipoprotein E (APOE) were selected for further study. All images, including heat maps, circular graphs, bubble diagrams, Sankey diagrams, and Venn diagrams, were generated using R packages and the OmicShare online platform (https://www.omicshare.com/tools/, accessed on 25 July 2025), as described previously [24]. Protein–protein interaction (PPI) networks of the DEPs and GO terms were constructed using STRING v11.5, Cytoscape 3.7.1, and Clue GO software (Cytoscape Consortium, San Francisco, CA, USA) [25,26]. Figures were generated using Adobe Illustrator 2022 (Adobe, San Jose, CA, USA) and MedPeer (MedPeer^®^, Beijing, China).

### 2.5. Hematoxylin and Eosin (H&E) Staining

Fixed PGs were embedded in paraffin (Solarbio, Beijing, China) and cut into 4–5 µm sections using a microtome (Leica, Wetzlar, Germany). H&E staining was performed as previously described. Briefly, after deparaffinization and dehydration, sections were stained using an H&E staining solution (Servicebio, Wuhan, China). After sealing with neutral resin (Solarbio), the sections were observed and imaged using an upright optical microscope equipped with an imaging system (Nikon, Tokyo, Japan). All staining assays were performed at least in triplicate.

### 2.6. Immunohistochemistry (IHC) and Immunofluorescence (IF) Staining

After antigen retrieval using citrate buffer (Servicebio, Wuhan, China), the sections were treated with 3.0% hydrogen peroxide (Servicebio, Wuhan, China) and blocked with 5.0% donkey serum (Solarbio, Beijing, China). Sections were incubated with rabbit anti-APOE (1:200; Cat No. 18254-1-AP, Proteintech, Wuhan, China) and anti-AR (1:50; sc-390429, Santa Cruz Biotechnology, Dallas, TX, USA) primary antibodies at different dilution ratios, as previously described. For IHC, sections were incubated with a secondary antibody (1:500, biotinylated rabbit/mouse IgG; Servicebio, Wuhan, China), and chromogenic reactions were performed using an ABC staining kit (Boster, Wuhan, China) and DAB substrate kit (Solarbio, Beijing, China), as described previously [17]. PBS was used in place of primary antibodies for the negative control group. For IF, sections were incubated successively with different primary antibodies, including anti-APOE, anti-AR, and anti-cytokeratin 7 (CK7, a biomarker for epithelial cells; 1:100; GB11225-100, Servicebio, Wuhan, China) and labeled with different colors of immunoglobulin G (Servicebio, Wuhan, China). Nuclei were stained with 10 µg/mL DAPI (Solarbio, Beijing, China). Sections were observed and imaged using a Nikon microscope (Tokyo, Japan) or an Olympus fluorescence microscope (Tokyo, Japan). After selecting 10 random views for each section, relative expression levels were measured using Image Pro Plus 6.0 software (https://mediacy.com/image-pro/; Media Cybernetics, Rockville, MD, USA; accessed on 15 May 2025). All immunostaining assays and measurements were performed in triplicate.

### 2.7. RNA Extraction, cDNA Synthesis and qRT-PCR

Total RNA was extracted from PGs (20 mg) in the Exp control and experimental groups using Trizol™ Reagent (Thermo Fisher, MA, USA) according to the manufacturer’s instructions [14,17]. RNA integrity was evaluated using 1% denaturing formaldehyde agarose gel electrophoresis (Biowest Regular Agarose, Castropol, Spain). RNA quantity and purity were assessed using a NanoDrop-8000 spectrophotometer (Thermo Fisher Scientific, Waltham, MA, USA). Total RNA (1 μg) was subjected to reverse transcription to single-stranded cDNA using the PrimeScript™ RT reagent kit (Accurate Biology, Changsha, China), as previously described [18,23]. Primers for *APOE* and *AR* (Supplementary Table S1) were designed using Premier software v5.0 (https://www.premierbiosoft.com/; PREMIER Biosoft, San Francisco, CA, USA; accessed on 25 May 2025) and synthesized by Qinke Biotech (Shanxi, China). qRT-PCR was performed on a LightCycler^®^ 96 real-time PCR system (Rocher, Basel, Switzerland) using a SYBR green master mix kit (Accurate Biology, Changsha, China), following the manufacturer’s instructions. All PCR procedures and calculations were performed as previously described [27]. *β-Actin* was used as the endogenous control. All qRT-PCR assays were performed at least in triplicate.

### 2.8. Western Blot

Total proteins were extracted from PGs (100 mg) in the control and experimental groups using a RIPA kit (Solarbio, Beijing, China) and then quantified using a BCA kit (Boster, Wuhan, China), according to the manufacturer’s instructions, as previously described [17,18]. After mixing with loading buffer (Solarbio, Beijing, China), the samples (50 µg) were used to examine APOE and AR expression levels in PGs from the control and experimental groups, as described previously [18]. Blots were visualized using ECL (Solarbio, Beijing, China) and analyzed using Image Pro Plus 6.0. β-Actin expression was used as an endogenous control, and APOE and AR expression levels in the control group were used as a control. All immunoblot assays were performed in triplicate.

### 2.9. Statistical Analysis

Unless otherwise stated, all statistical measurements for the groups are presented as the mean ± SEM. All statistical analyses were performed using SPSS software (v27.0; SPSS Inc., Chicago, IL, USA). Figures were generated using GraphPad Prism 8.0 (GraphPad Software Inc., San Diego, CA, USA). Differences in mean values between groups were analyzed using Student’s *t*-test (between two groups) or one-way ANOVA. * *p* < 0.05 and ** *p* < 0.01 indicate a statistically significant difference.

## 3. Results

### 3.1. T-CHO and Testosterone Concentrations in Camel PGs During the Breeding Seasons

**Figure 1:**
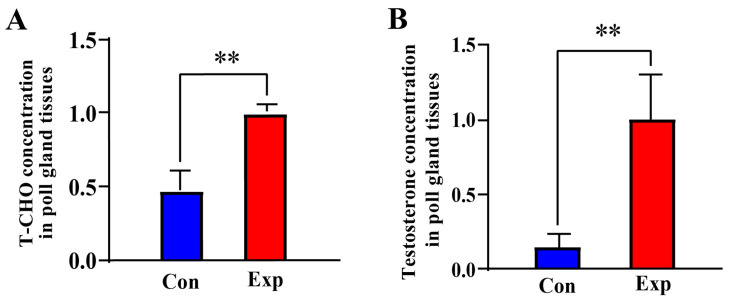
T-CHO and testosterone concentrations detected in camel PGs. (**A**) T-CHO content; (**B**) testosterone content Con, control group; Exp, experimental group. ** *p* < 0.01.

Compared to the control group, the T-CHO and testosterone concentrations in PGs of the experimental group were increased by almost two-fold (*p* < 0.01) and almost five-fold (*p* < 0.01), respectively (Figure 1A,B). These results indicate that T-CHO and testosterone concentrations were positively correlated in camel PGs during the breeding season.

### 3.2. Identification of Steroid-Associated DEMs from Metabolomics in Camel PGs

**Figure 2:**
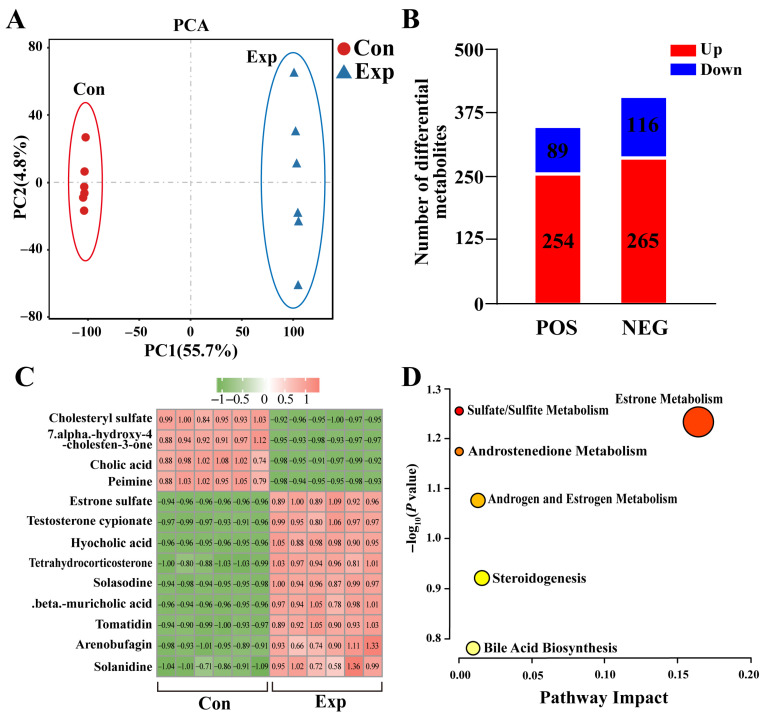
Identification of steroid-associated differentially expressed metabolites (DEMs) from metabolomics of camel PGs. (**A**) Principal component analysis of metabolomic test samples. (**B**) DEMs detected in positive (POS) and negative (NEG) modes of metabolomics. (**C**) Steroid-associated DEMs identified using metabolomics. (**D**) Pathway enrichment analysis of steroid-associated DEMs. Con, control group; Exp, experimental group.

The metabolomics results showed significant differences in metabolites in camel PGs at different phases (Figure 2A). A total of 343 DEMs (254 upregulated and 89 downregulated) were detected in positive-ion mode, whereas 381 DEMs (365 upregulated and 116 downregulated) were detected in negative-ion mode (Figure 2B). Combining the differential metabolites from both modalities resulted in a total of 13 DEMs associated with steroids (nine upregulated and four downregulated), such as cholic acid, estrone sulfate, and testosterone cypionate (Figure 2C). Enrichment analysis showed that these DEMs were mainly involved in steroid-hormone-related pathways (Figure 2D). These results suggest that the metabolic capacity of steroid-related substances is significantly enhanced in PGs during the peak breeding season.

### 3.3. Identification of Candidate DEPs Associated with Steroids Based on the GO Terms of DIA Proteomics

**Figure 3:**
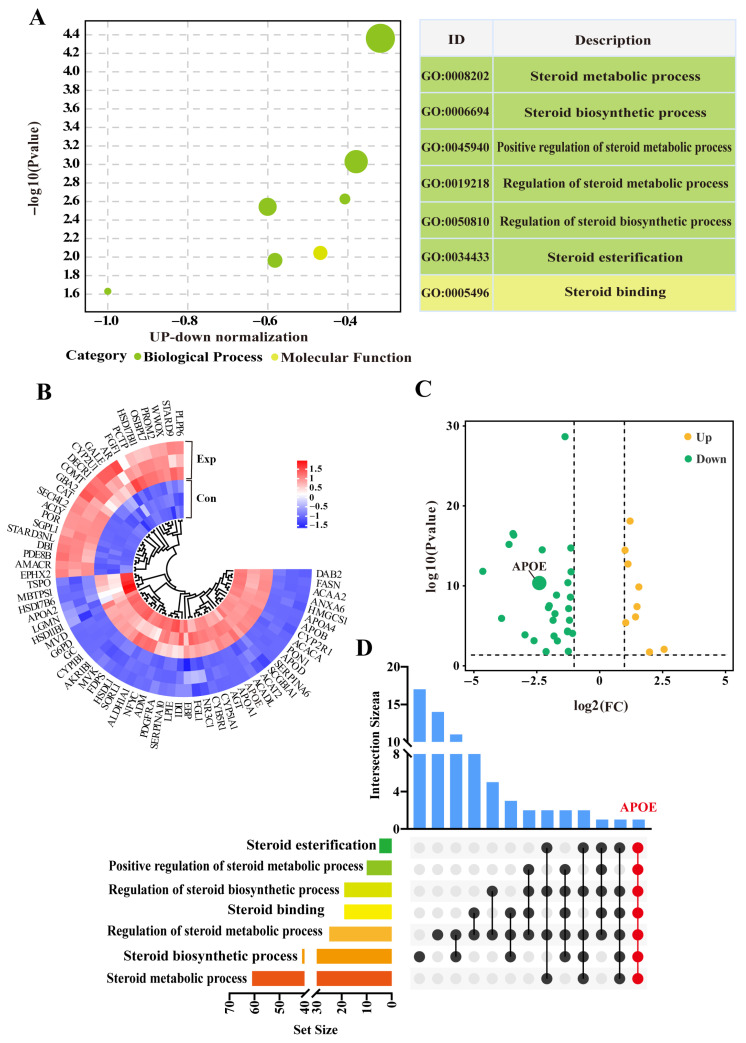
Identification of steroid-related candidate differentially expressed proteins (DEPs) based on GO terms. (**A**) Enrichment analysis showing seven GO terms associated with steroids based on DIA proteomics. (**B**) Volcano plot analysis of DEPs across seven enriched GO terms. (**C**) Heatmap clustering of DEPs across seven enriched GO terms. (**D**) Upset diagram analysis of GO terms and DEPs. Con, control group; Exp, experimental group.

Seven GO terms, including six biological process terms and one molecular function term, were screened from the DIA data (Supplementary Table S2). These GO terms were mainly related to steroid metabolism, biosynthesis, and regulation, particularly steroid esterification (Figure 3A). After removing duplicate DEPs, a total of 69 DEPs (31 downregulated and 12 upregulated) were identified within the GO terms (Figure 3B). The heatmap showed relatively consistent expression of these DEPs in three replicates of the control group, whereas their expression patterns differed significantly between control and experimental groups (Figure 2C). The UpSet Venn diagram demonstrated that APOE, a unique DEP, was shared among these GO terms (Figure 2D). These results highlight these 69 DEPs, and especially APOE, as playing important roles in steroid synthesis and transport in camel PGs, during the breeding season.

### 3.4. Identification of Pathways Interacting with APOE

**Figure 4:**
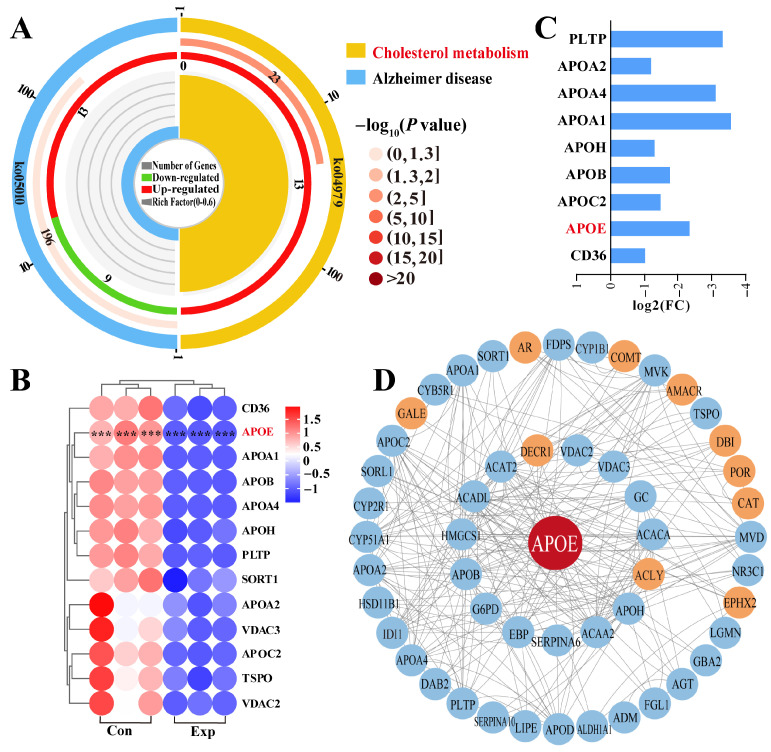
Identification of pathways and DEPs interacting with APOE and enrichment analysis. (**A**) Enrichment analysis identifying two pathways associated with APOE. (**B**) Relative expression levels of DEPs quantified by DIA proteomics. (**C**) Heatmap of 13 DEPs in the cholesterol metabolism pathway. (**D**) Protein–protein interaction network of DEPs identified from GO terms and pathways enrichment analysis. Con, control group; Exp, experimental group. *** represents *p* < 0.001.

Pathway enrichment analysis revealed that APOE was involved in two pathways, most notably the cholesterol metabolism pathway, which showed significant differences (Figure 4A). Therefore, we focused on cholesterol metabolism (Supplementary Table S3), which involved 13 DEPs (Figure 4A). The heatmap showed that these 13 DEPs were differentially expressed between the control and experimental groups, especially in the control group, which showed significantly different intra-group variation in expression levels (Figure 4B). Notably, compared to the control group, all 13 DEPs were downregulated in the experimental group according to log2 (fold change) values (Figure 4C). Taking APOE as the core of the interaction, we constructed a PPI network of the 69 DEPs identified from the GO terms and 13 DEPs identified from pathway enrichment analysis (Figure 4D). The results showed that 11 DEPs directly interacted with APOE, and seven DEPs were connected to APOE by a single node. Meanwhile, 33 DEPs showed two or more interactions with APOE. These findings suggest that the functions of APOE and related DEPs are associated with steroid synthesis and transport, which affect steroid hormone synthesis via cholesterol metabolism.

### 3.5. Distribution, Expression Patterns, and Co-Localization Analysis of APOE and AR in Camel PGs

**Figure 5:**
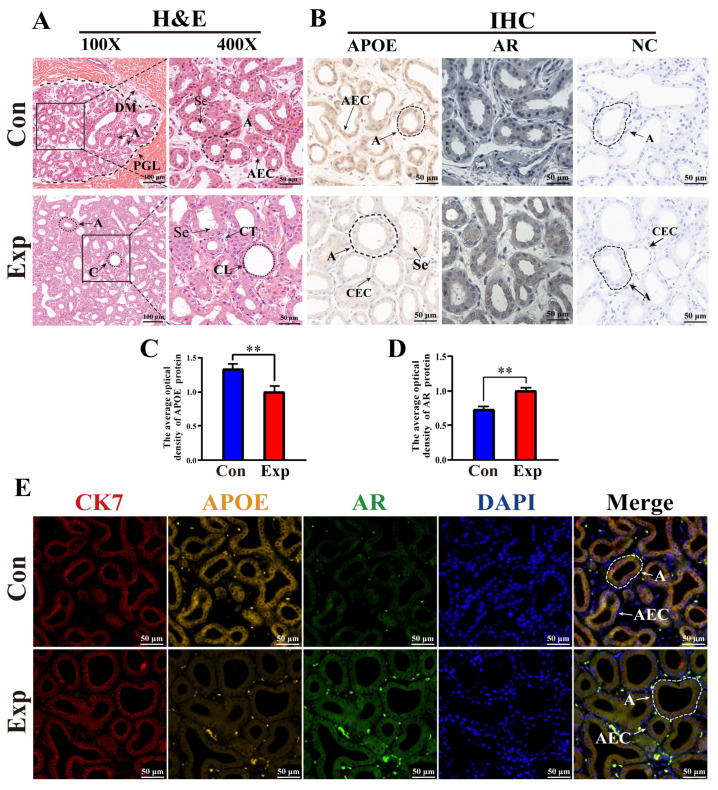
Distribution, expression patterns, and co-localization analysis of APOE and AR in camel PGs. (**A**) Histological observation of PGs performed using H&E staining. (**B**) APOE and AR protein distribution assessed via immunohistochemical (IHC) analysis. (**C**,**D**) Optical density of APOE and AR proteins, respectively, quantified from the IHC staining results. (**E**) Immunofluorescence staining results of poll glands for anti-APOE, AR, and CK7 proteins. Abbreviations: A, alveolar; C, catheter; DM, dermal muscles; PGL, poll gland lumen; Se, secreta; CT, connective tissues; CL, catheter lumen; AEC, alveolar epithelial cell; CEC, catheter epithelial cell. Con, control group; Exp, experimental group. ** *p* < 0.01.

According to H&E staining, PGs in the control group were characterized by lobules surrounded by connective tissue and muscle fibers with clear boundaries, a small number of acini, and thin acinar walls. PGs in the experimental group were characterized by a much higher number of acini, thickened acinar walls, flattened epithelial cells, clear ductal structures lobules filled with acini and ducts, and increased secretions in the acini (Figure 5A). IHC staining showed that immunopositive signals of APOE and AR proteins were mainly expressed in acinar epithelial cells, with different degrees of staining between the two groups. No positive staining for APOE or AR was observed in the negative control group (Figure 5B). Compared to the control group, the average optical density of APOE protein was significantly decreased in the experimental group, whereas AR protein levels were significantly upregulated in the experimental group (Figure 5C,D). Positive signals for APOE, AR, and CK7 proteins were present in the cytoplasm of the acinar epithelium in the PGs of both groups, as evidenced by the IF staining results (Figure 5E). These results indicate that the function of APOE protein is closely related to epithelial cells.

### 3.6. Relative Protein and mRNA Expression Levels of APOE and AR in Camel PGs

**Figure 6:**
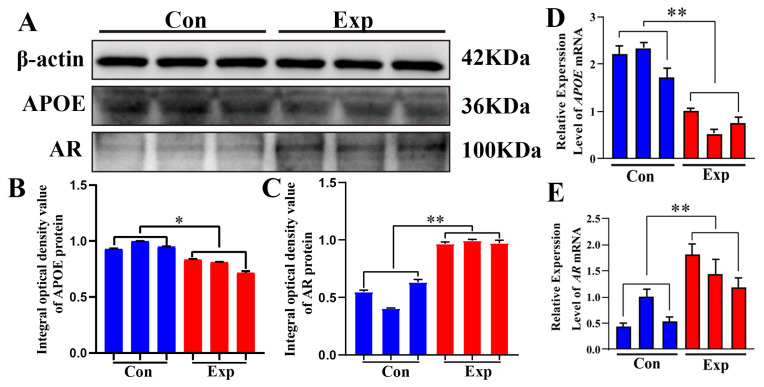
Relative mRNA and protein expression levels of *APOE* and *AR* in camel PGs. (**A**) APOE, AR, and β-actin proteins detected in the PGs of peak-estrus and early-estrus groups using Western blot. Complete Western blots are shown in Supplementary Figures S1–S3. (**B**,**C**) Relative integrated optical density values of APOE and AR proteins in the PGs of both groups. (**D**,**E**) *APOE* and *AR* mRNA expression levels in the PGs of both groups. β-actin was used as an endogenous control. Con, control group; Exp, experimental group. * *p* < 0.05, ** *p* < 0.01.

APOE and AR proteins were detected in both groups (Figure 6A). Compared to the control group, APOE protein was significantly downregulated and AR protein was significantly upregulated in the experimental group (Figure 6B,C). Compared to the control group, the relative mRNA expression levels of *APOE* and *AR* were significantly downregulated and significantly upregulated, respectively, in the experimental group (Figure 6D,E). The strong decrease in APOE protein levels observed in the experimental group contrasts with the changes observed in cholesterol and steroid hormone levels, indicating that APOE may play a negative regulatory role in the synthesis of steroid hormones.

## 4. Discussion

Bactrian camels, categorized as livestock with low reproductive capacity, exhibit significant disparities in their breeding season and mating phases compared with other livestock [14]. PGs release a viscous fluid containing pheromone-like substances that signal to female camels to induce mating, which is closely linked to male camel breeding behavior. The breeding season of mammals is governed by the hypothalamic–pituitary–gonadal axis through hormonal regulation [28]. The coordinated involvement of reproductive-related hormones such as gonadotropins, catecholamines, and steroid hormones (primarily testosterone, dihydrotestosterone, and androstenedione) is required for male reproductive activity [29]. Cholesterol, which serves as an exclusive precursor for steroid hormone synthesis, undergoes intricate transport mechanisms and biochemical reactions that are ultimately converted into steroid hormones in the interstitial cells of the testes or adrenal cortex [30]. Our CHO and testosterone measurements confirmed these findings. Thus, understanding the regulatory mechanisms of cholesterol and steroid hormones in PGs is important for improving reproductive performance.

In this study, steroid-associated DEMs and DEPs in PGs of male Bactrian camels were analyzed using metabolomics and DIA proteomics during the peak and early breeding seasons. Various steroid-related DEMs, including testosterone, were upregulated during the peak breeding season. Proteomics identified seven GO terms directly related to steroids; APOE proteins were involved in all steroid-associated GO terms, revealing APOE as an important candidate DEP. Integrated metabolomic and proteomic analyses revealed the coordinated regulation of metabolites and proteins involved in steroidogenesis. Several steroid-related metabolites, including testosterone and cholesterol derivatives, were elevated during the peak breeding season, indicating enhanced steroid biosynthesis. APOE was downregulated whereas AR was upregulated, suggesting that reduced APOE-mediated cholesterol efflux and increased AR activity promote cholesterol utilization for steroid hormone synthesis. Thus, APOE may serve as a molecular link connecting metabolite and protein-level regulation of steroidogenesis in male Bactrian camels. Previous studies have implicated APOE as a genetic risk factor for Alzheimer’s disease [31,32,33], mainly owing to its role in lipid and cholesterol transport in the brain. In camels, however, APOE appears to play a similar functional role in cholesterol metabolism within the poll glands, contributing to the regulation of steroid hormone synthesis during the breeding season. KEGG pathway analysis revealed that APOE was involved in cholesterol metabolism. PPI analysis confirmed that candidate DEPs within the relevant GO terms and pathways interacted with APOE and steroid hormone synthesis. The APOE protein, as a major lipid transport protein, differs from most lipid transport proteins by performing its functions through autocrine or paracrine mechanisms that promote the local redistribution of cholesterol and influence intracellular cholesterol levels [34,35]. The binding of high-density lipoprotein and APOE facilitates cholesterol efflux, which leads to decreased intracellular cholesterol content in hepatic cells [34]. High-density lipoprotein acts as an extracellular receptor for APOE, facilitating its recycling and reuse outside cells [36]. The retained cholesterol is primarily utilized for membrane construction and steroid hormone synthesis in the cells. These findings indicate that APOE is closely related to cholesterol metabolism in animals.

H&E staining and IHC results revealed a significant increase in acinar and ductal epithelial cells in the experimental group compared with those in the control group, indicating substance exchange and secretion in PGs during the peak breeding season. IHC and IF results showed that APOE, AR, and CK7 proteins were co-localized in the cytoplasm of the acinar epithelium in PGs. APOE can directly influence the levels and distribution of cholesterol to indirectly impair steroid hormone synthesis [37]. AR activation upregulates the activity of sterol regulatory element-binding protein, which promotes cholesterol synthesis and expression of the low-density lipoprotein receptor (LDLR), and increases the cellular uptake of cholesterol [38]. AR regulation of LDLR expression by AR may affect the lipid metabolism pathway mediated by APOE because APOE clears lipoproteins through LDLR family receptors. Moreover, significant downregulation of APOE protein expression was observed in PGs during the peak breeding season. qRT-PCR and Western blot results confirmed that *APOE* mRNA and protein levels were lower in the peak breeding season than in the early breeding season, indicating a weakened cholesterol efflux function of APOE in PGs during the peak breeding season. In contrast, *AR* mRNA and protein levels were higher in the peak breeding season than in the early breeding season, suggesting that more cholesterol was used for steroid hormone synthesis. Thus, a negative correlation exists between PG cholesterol and testosterone levels and APOE protein expression. Previous studies have reported that a class of miRNAs in APOE carriers can inhibit de novo cholesterol synthesis [35,39]. APOE overexpression can also significantly decrease de novo cholesterol synthesis and esterification [40,41].

**Figure 7:**
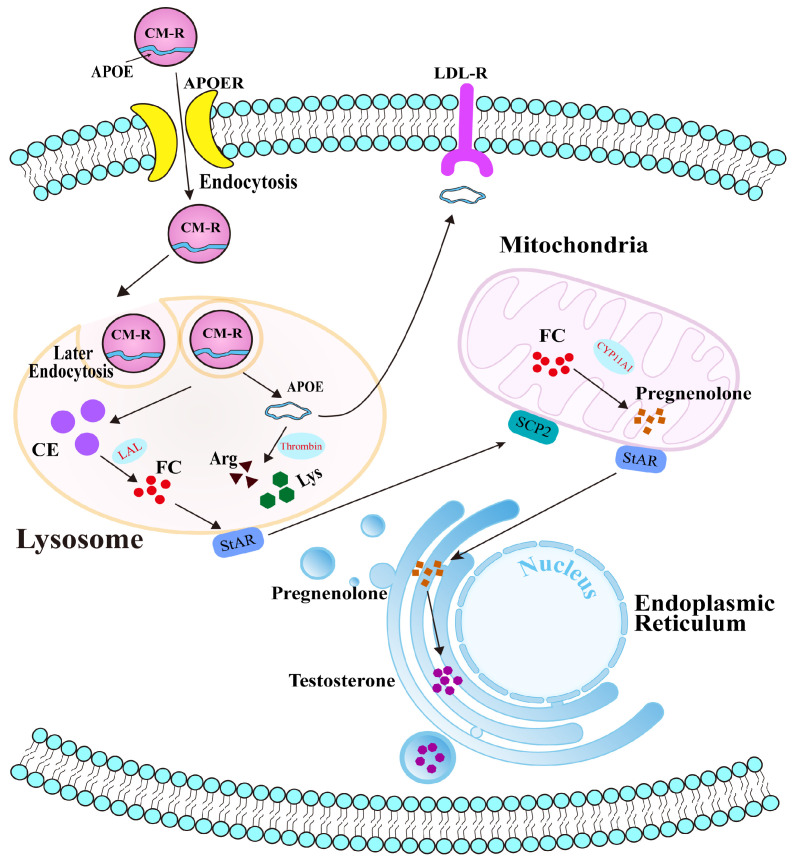
Potential molecular mechanism of APOE-mediated cholesterol transport in the PGs of male Bactrian camels.

Collectively, our findings reveal the potential molecular mechanism of APOE in cholesterol transport and steroidogenesis in the PGs of Bactrian camels during the breeding season (Figure 7). Under physiological conditions, APOE typically binds to triglycerides to form chylomicrons, which are subsequently hydrolyzed by LPL to form chylomicron remnants (CM-R) that enter cells via APOER-mediated endocytosis. CM-R undergo secondary endocytosis and enter lysosomes, where they are degraded, releasing cholesterol esters and freeing APOE within the cytoplasm. Although some APOE is returned to the extracellular space, the remainder is cleaved by thrombin into arginine and lysine, which participate in other physiological processes. During the early breeding phase, APOE facilitates the transport of CM-R into cells. Decreased APOE protein expression therefore results in cholesterol accumulation and accelerated steroid hormone synthesis in PGs.

This study has some limitations. First, the relatively small sample size may limit the statistical power of our findings. Nevertheless, the consistent biological patterns observed among individuals, together with the multi-omics validation, support the reliability and robustness of our conclusions. In addition, further studies employing both in vitro and in vivo models are required to functionally validate the roles of APOE and AR in cholesterol accumulation and steroid hormone synthesis. Nonetheless, this study highlights the potential role of APOE in steroid hormone synthesis. Compared with other livestock species such as cattle and pigs, Bactrian camels exhibit a shorter and more seasonally restricted breeding period, largely constrained by their low reproductive efficiency and harsh environmental adaptations. Here, we propose APOE-mediated cholesterol metabolism and AR-regulated steroidogenesis as potential unique regulatory pathways that contribute to the reproductive physiology of camels. This study not only expands our understanding of camel reproductive biology but can also inform breeding management strategies; for example, by optimizing the timing of mating or developing molecular markers to improve reproductive efficiency in camels.

## 5. Conclusions

In this study, we found that T-CHO and testosterone concentrations differed significantly between male Bactrian camels from the early breeding season and peak breeding season. We identified a total of 13 steroid-associated DEMs and seven GO terms including 69 DEPs from metabolomic and DIA proteomic analyses of PGs. APOE was identified as a core DEP involved in cholesterol metabolism and steroid hormone synthesis. APOE and AR proteins were mainly co-localized in the cytoplasm of acinar and ductal epithelial cells of PGs. Compared to the early breeding season group, the peak breeding season group exhibited significant downregulation and significant upregulation of the mRNA and protein levels of *APOE* and *AR.* Our findings indicate that APOE-mediated cholesterol metabolism plays an important role in steroid hormone synthesis in PGs during camel reproduction. This research provides a theoretical basis for understanding the reproductive mechanisms of Bactrian camels.

## Data Availability

The data that support the findings of this study are available from the corresponding author upon reasonable request.

## Supplementary Materials

The following supporting information can be downloaded at: https://www.mdpi.com/article/10.3390/ani15213147/s1. Table S1: qRT-PCR primer sequence. Table S2: GO analysis from DIA proteomics. Table S3: Pathway analysis from DIA proteomics. Figures S1–S3: Whole Western blots of β-actin, APO1, and AR, respectively.

## Abbreviations

The following abbreviations are used in this manuscript:

PGs	Poll gland tissues
HSD3β	3β-hydroxysteroid dehydrogenase
DEMs	Differentially expressed metabolites
DEPs	Differentially expressed proteins
T-CHO	Total cholesterol
KEGG	Kyoto Encyclopedia of Genes and Genomes
DIA	Data-independent acquisition
GO	Gene Ontology
APOE	Apolipoprotein E
PPI	Protein–protein interaction
H&E	Hematoxylin and eosin
IHC	Immunohistochemistry
IF	Immunofluorescence
AR	Androgen receptor
CK7	Cytokeratin 7
CM-R	Chylomicron remnants
