# Supplementary material for: Multi-Omics Profiling Identifies Apolipoprotein E as an Important Regulator of Steroidogenesis in Bactrian Camel Poll Glands During the Breeding Season
Source: Animals (Basel). 2025 Oct 30;15(21):3147. doi: 10.3390/ani15213147 (PMC12607609; doi:10.3390/ani15213147)
Supplement: Supplementary file 1 [file animals-15-03147-s001.zip › Supplementary Materials/Table S1 (qRT-PCR primer sequence).pdf]

Table S1 qRT-PCR primer sequence

Gene name	Accession NO.	Primer Sequence 5'-3'	Product Length
β-actin	XM-010965866.1	F: TCCCTGGAGAAGAGCTACGA<br>R: CAGCACCGTGTTAGCGTAGA	181 bp
APOE	XM_010946790.2	F: GATGAAAGCTCTGTGGGTTG<br>R: GCAGGTAATCCAGAGACGA	182 bp
AR	XM_010973649.2	F: TCGCAGCCTTACTCTCTAGC<br>R: AACACCATAAGCCCCATCCA	193 bp

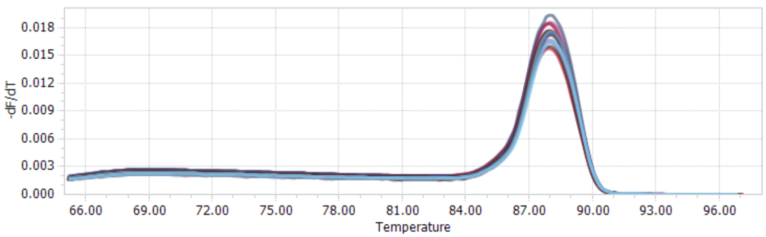

Dissolution curve of *β-actin* Primer

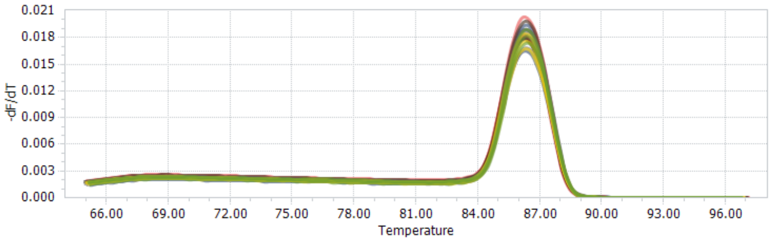

Dissolution curve of *APOE* Primer

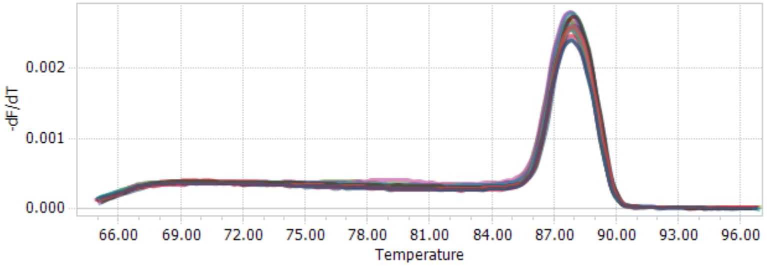

Dissolution curve of *AR* Primer
