# Supplementary figures and images for: Multi-Omics Profiling Identifies Apolipoprotein E as an Important Regulator of Steroidogenesis in Bactrian Camel Poll Glands During the Breeding Season
Source: Animals (Basel). 2025 Oct 30;15(21):3147. doi: 10.3390/ani15213147 (PMC12607609; doi:10.3390/ani15213147)

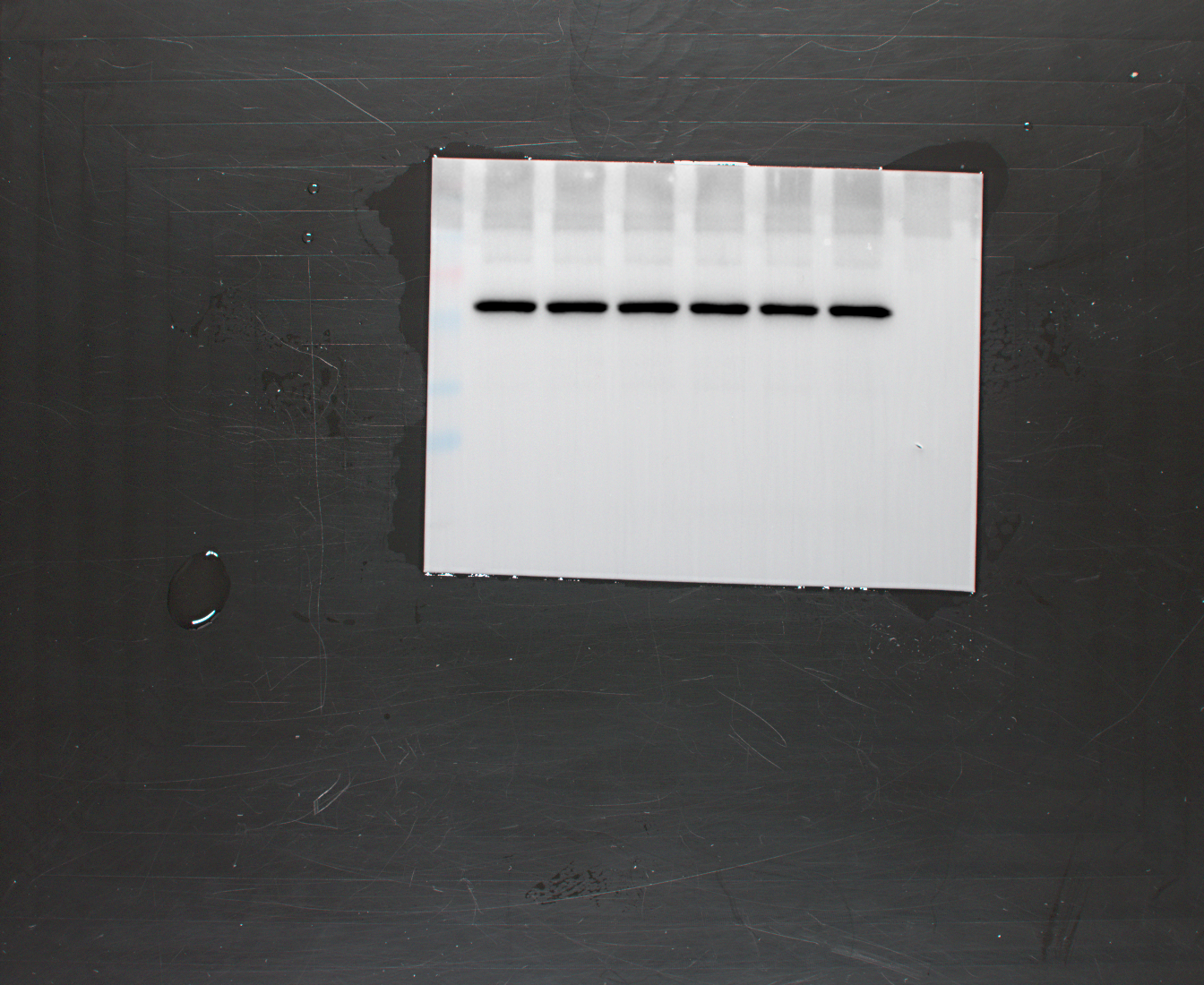

Supplement: Supplementary file 1 [file animals-15-03147-s001.zip › Supplementary Materials/Figure S1 β-actin Blot.Tif]

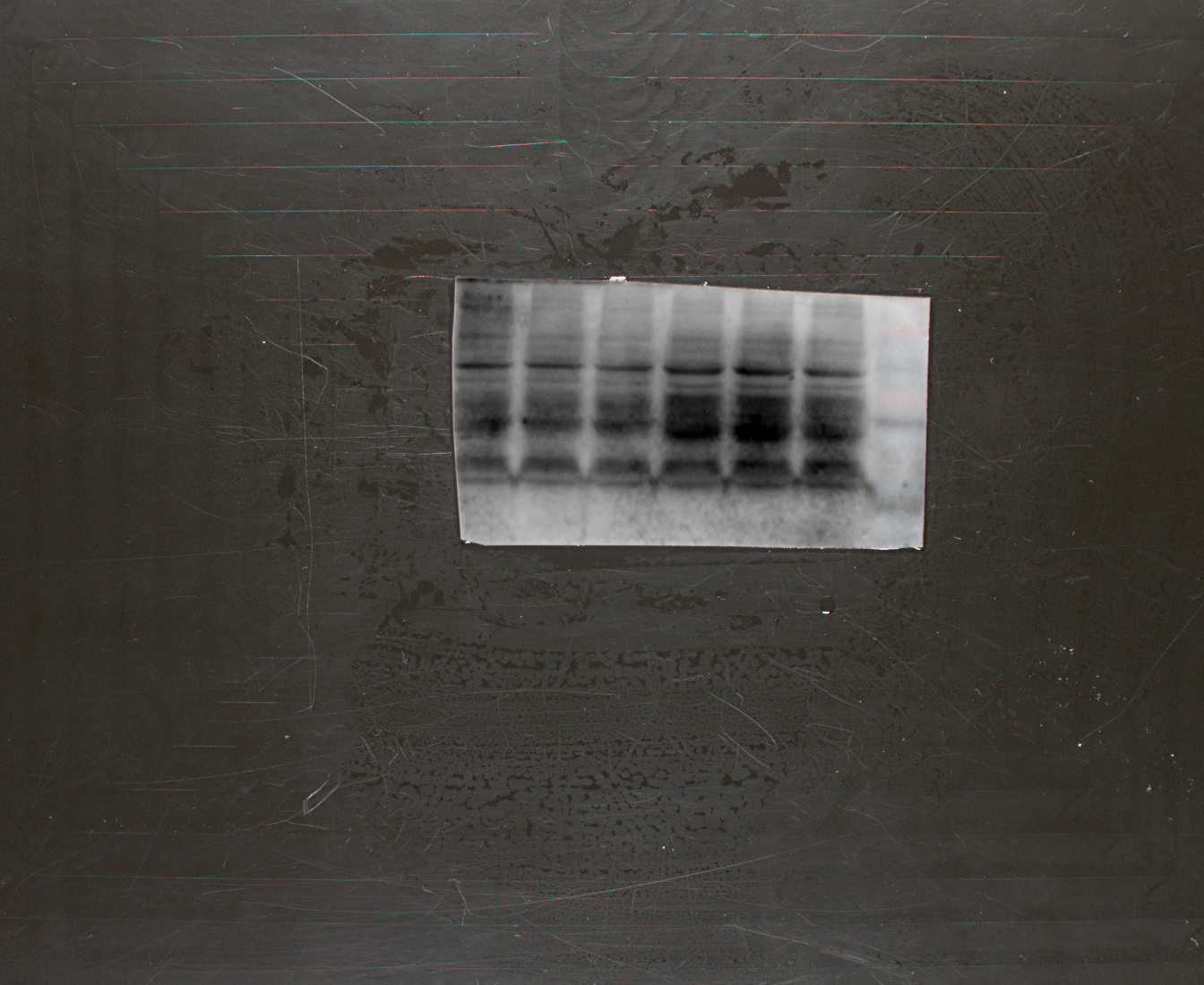

Supplement: Supplementary file 1 [file animals-15-03147-s001.zip › Supplementary Materials/Figure S2 APOE Blot.Tif]

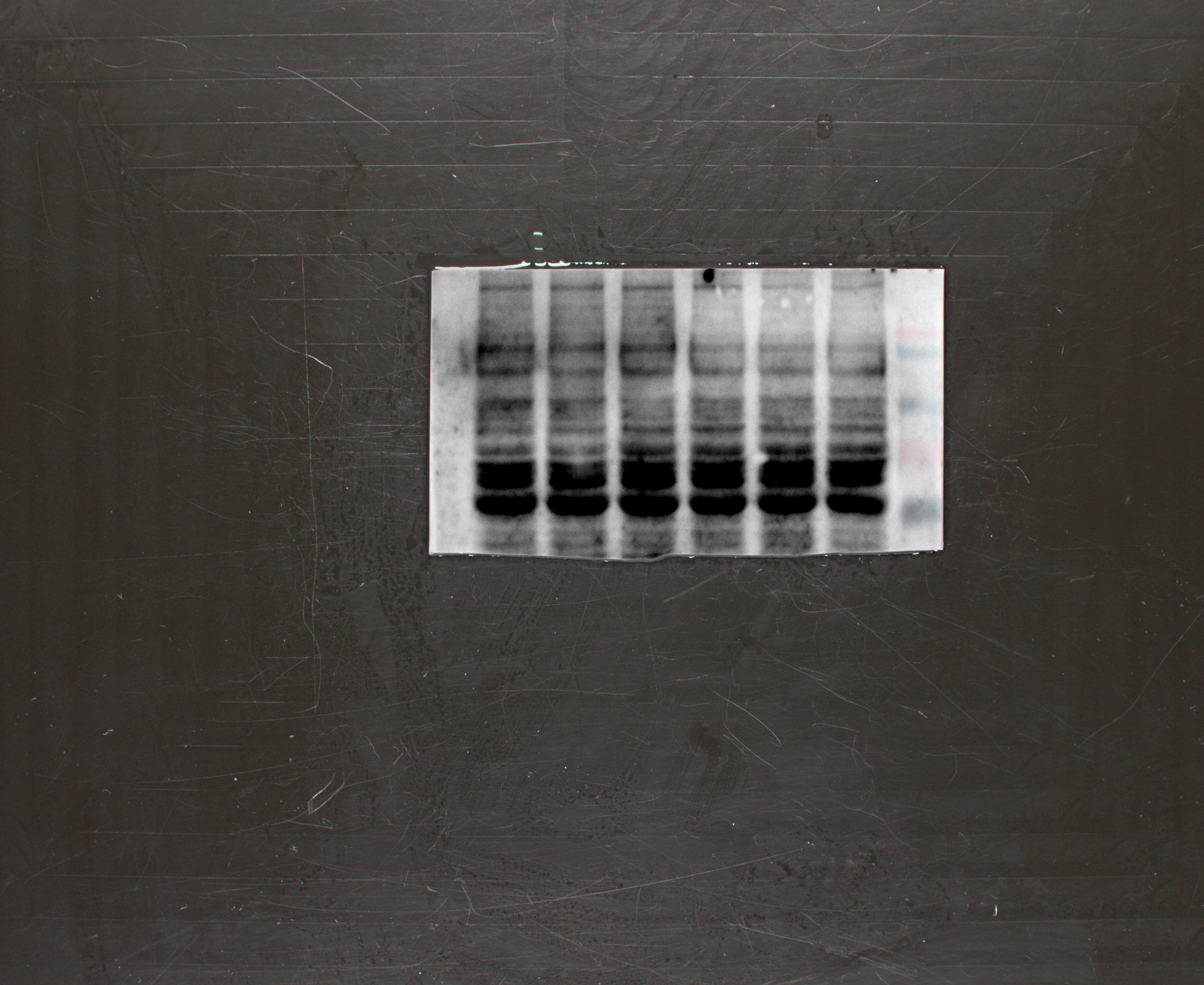

Supplement: Supplementary file 1 [file animals-15-03147-s001.zip › Supplementary Materials/Figure S3 AR Blot.tif]
